# Social Preference Deficits in Juvenile Zebrafish Induced by Early Chronic Exposure to Sodium Valproate

**DOI:** 10.3389/fnbeh.2016.00201

**Published:** 2016-10-20

**Authors:** Xiuyun Liu, Yinglan Zhang, Jia Lin, Qiaoxi Xia, Ning Guo, Qiang Li

**Affiliations:** ^1^Translational Medical Center for Development and Disease, Shanghai Key Laboratory of Birth Defect, Institute of Pediatrics, Children's Hospital of Fudan UniversityShanghai, China; ^2^Department of Life Sciences, Anhui Science and Technology UniversityAnhui, China; ^3^Center for Chinese Medical Therapy and Systems Biology, Shanghai University of Traditional Chinese MedicineShanghai, China

**Keywords:** sodium valproate, zebrafish, behavior, social preference, locomotor activity, anxiety

## Abstract

Prenatal exposure to sodium valproate (VPA), a widely used anti-epileptic drug, is related to a series of dysfunctions, such as deficits in language and communication. Clinical and animal studies have indicated that the effects of VPA are related to the concentration and to the exposure window, while the neurobehavioral effects of VPA have received limited research attention. In the current study, to analyze the neurobehavioral effects of VPA, zebrafish at 24 h post-fertilization (hpf) were treated with early chronic exposure to 20 μM VPA for 7 h per day for 6 days or with early acute exposure to 100 μM VPA for 7 h. A battery of behavioral screenings was conducted at 1 month of age to investigate social preference, locomotor activity, anxiety, and behavioral response to light change. A social preference deficit was only observed in animals with chronic VPA exposure. Acute VPA exposure induced a change in the locomotor activity, while chronic VPA exposure did not affect locomotor activity. Neither exposure procedure influenced anxiety or the behavioral response to light change. These results suggested that VPA has the potential to affect some behaviors in zebrafish, such as social behavior and the locomotor activity, and that the effects were closely related to the concentration and the exposure window. Additionally, social preference seemed to be independent from other simple behaviors.

## Introduction

Zebrafish, a vertebrate animal, offers several advantages for analyzing neurobehaviors (Wang et al., 2014). To start with, its strong reproductivity and small size make it possible to test detailed dose-response and window-response relationships with low costs in terms of time and money. Next, zebrafish develop rapidly. The neural tube is formed at 12 hpf (Hjorth and Key, 2002; Chapouton and Godinho, 2010) and zebrafish develop into free-swimming larvae at 48 hpf (Kalueff et al., 2013), which makes it convenient and time-saving to do research in this model. In addition, zebrafish share similar brain structures with mammals. The counterparts of many brain regions found in the developing mammalian brain are also observed in the developing zebrafish brain including the cortex, hippocampus, amygdala, habenula, thalamus, and cerebellum, which makes the zebrafish an ideal model to study neuropsychological diseases (Wullimann and Mueller, 2004; Mueller et al., 2008; Wullimann, 2009). Furthermore, zebrafish also exhibit numerous simple and complex neurobehaviors, including spontaneous swimming, startle responses, and learning (Farrell et al., 2011; Tierney, 2011; Ingebretson and Masino, 2013; Kalueff et al., 2014). Multiple simple behaviors have been detected and analyzed as early as 5 days post-fertilization (dpf), such as the locomotor activity, thigmotaxis, and the startle response (Liu et al., 2016), while complex behaviors such as learning, memory, and social behaviors can be detected later (Engeszer et al., 2004; Wong et al., 2010; Miller and Gerlai, 2012; Fernandes et al., 2016). Finally, some behavioral assays for complex behaviors are easier to perform in zebrafish, such as shoaling (Maaswinkel et al., 2013; Mahabir et al., 2013). Therefore, zebrafish represents an animal model that is beneficial for understanding the mechanisms that underlie various behaviors.

Social behaviors, such as the preference to be close to and mimic conspecifics, are common to humans (Xiao et al., 2014), many other mammals (Ferrari et al., 2006) and non-mammalian vertebrates (Engeszer et al., 2004, 2007; Mooney, 2014). Zebrafish are a highly social species. Adult zebrafish exhibit a range of social behaviors, such as shoaling, schooling and aggression (Green et al., 2012; Miller and Gerlai, 2012; Jones and Norton, 2015), while larval zebrafish do not obviously exhibit those social behaviors. Social preference, preferring to be near conspecifics, is a foundation of other complex social behaviors, such as shoaling and schooling. Compared to shoaling and schooling, the assay for social preference is very simple and convenient. Thus, social preference has been studied as social behaviors commonly. SCH-23390, a D1-receptor antagonist, has been demonstrated to significantly reduce social preference in adult zebrafish (Scerbina et al., 2012). In addition, ibogaine-treated zebrafish displayed altered preference to conspecifics and altered shoaling behavior (Cachat et al., 2013). Furthermore, oxytocin (OT) and arginine-vasopressin (AVP) have been shown to increase social preference in zebrafish (Braida et al., 2012).

Valproic acid, a short-chain branched fatty acid, is an antagonist of sodium and calcium channels and has been used clinically in the treatment of epilepsy and bipolar disorder. However, clinical research has clearly demonstrated that VPA administration during pregnancy is accompanied by many risks, such as congenital malformations and other birth defects, developmental delay, reduced cognitive function, and more recently, increased risk of autism (Roullet et al., 2013). Additionally, the effects of VPA on behaviors have been studied in animal models. First, administration of VPA to BALB/C mice on postnatal day 14 induced increased locomotor activity and anxiety and decreased social behaviors (Pragnya et al., 2014). Rats receiving VPA on the embryonic day12.5 (E12.5) exhibited lower sensitivity to pain and higher sensitivity to nonpainful stimuli, as well as locomotor and repetitive/stereotypic-like hyperactivity combined with lower exploratory activity, increased anxiety, and decreased number of social behaviors (Schneider and Przewlocki, 2005; Schneider et al., 2007, 2008; Markram et al., 2008). Further, zebrafish exposed to VPA (48 μM) during the first 48 h of development displayed increased locomotor activity and anxiety at 6 dpf, increased anxiety and impaired social behavior at 70 dpf, and decreased anxiety at 120 dpf (Zimmermann et al., 2015). Bailey et al. exposed zebrafish to VPA at a series of concentrations (0.5, 5, 15, 30, and 50 μM) from 4 to 5 dpf and found that zebrafish treated with 15 μM VPA exhibited increased locomotor activity at 6 dpf and that zebrafish treated with 5 μM VPA exhibited impaired social behaviors in adulthood (Bailey et al., 2015). The neurobehavioral effects of VPA have been studied in animal models, and the results were dependent on the concentration and the exposure procedure. To clarify the relationship between the neurobehavioral effects of VPA and the exposure concentration and the exposure procedure, more research work are needed.

In the current work, two different exposure procedures were taken into consideration to analyze the neurobehavioral effects of VPA. Early chronic exposure to VPA at a low concentration and early acute exposure to VPA at a high concentration were studied. Both simple and complex behaviors were measured, including the locomotor activity, behavioral response to light change, anxiety, and social preference.

## Materials and methods

### Animals

Zebrafish eggs were obtained by random mating between sexually mature zebrafish (AB strain) and were kept with blue egg water in dishes. Collected eggs were inspected under a dissection microscope at 6 hpf, and those developing normally were selected. Eggs were randomly grouped into different exposure conditions (described below in Section Chemical Exposure) after 24 hpf and were raised in a transparent pc fry incubator at 28.5°C under a 14:10-h light:dark cycle (lights on at 08:00 a.m.). Larvae were allowed to develop under these conditions until 7 dpf. Then, the larvae were transferred to system water in 3 L tanks and fed with *Paramecium caudatum*. The larvae were moved into the commercial circulating rack system (ESEN EnvironScience) as soon as the establishment of fairy shrimp-eating behavior and were maintained until 1 month of age. The pH and the salt concentrations of the system water were monitored automatically, and they were also inspected periodically and manually. All of the animal experimental procedures complied with local and international regulations. All of the protocols were approved by the institutional animal care committee, Children's Hospital of Fudan University.

### Chemical exposure

Valproic acid sodium salt (P4543-10G, Sigma-Aldrich) was dissolved in a 500 mM stock solution with sterilized water and stored at −80°C. Before the experiments, VPA working solution was freshly diluted from stock solution to appropriate concentrations with zebrafish system water before the experiments.

Chronic exposure: beginning at 24 hpf, 60 larvae were exposed to 20 μM VPA in the transparent pc fry incubator for 7 h per day for 6 days.

Acute exposure: beginning at 24 hpf, 60 larvae were exposed to 100 μM VPA for 7 h.

Larvae in each group were inspected daily under a dissection microscope during exposure, and larvae with arrested development or obvious malformations were excluded.

**Figure 1:**
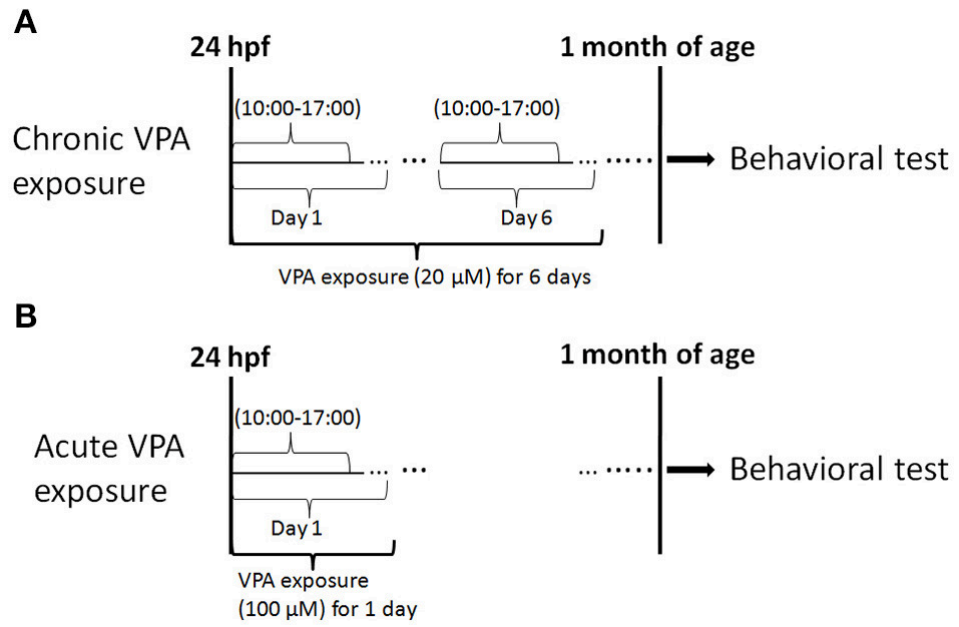
**Experimental procedure**. This experiment consisted of drug exposure and behavioral testing. **(A)** Chronic exposure to 20 μM VPA occurred for 7 h (starting at 10:00 a.m.) every day from 1 to 6 dpf and behavioral tests were performed at 1 month of age. **(B)** Acute exposure to 100 μM VPA was applied for 7 h at 1 dpf, and behavioral tests were performed at 1 month of age.

At the end of exposure, fish were carefully rinsed three times with fresh system water before transferring to normal rearing condition. See Figure 1 for a summary of the experimental design.

### Behavioral assessment

**Figure 2:**
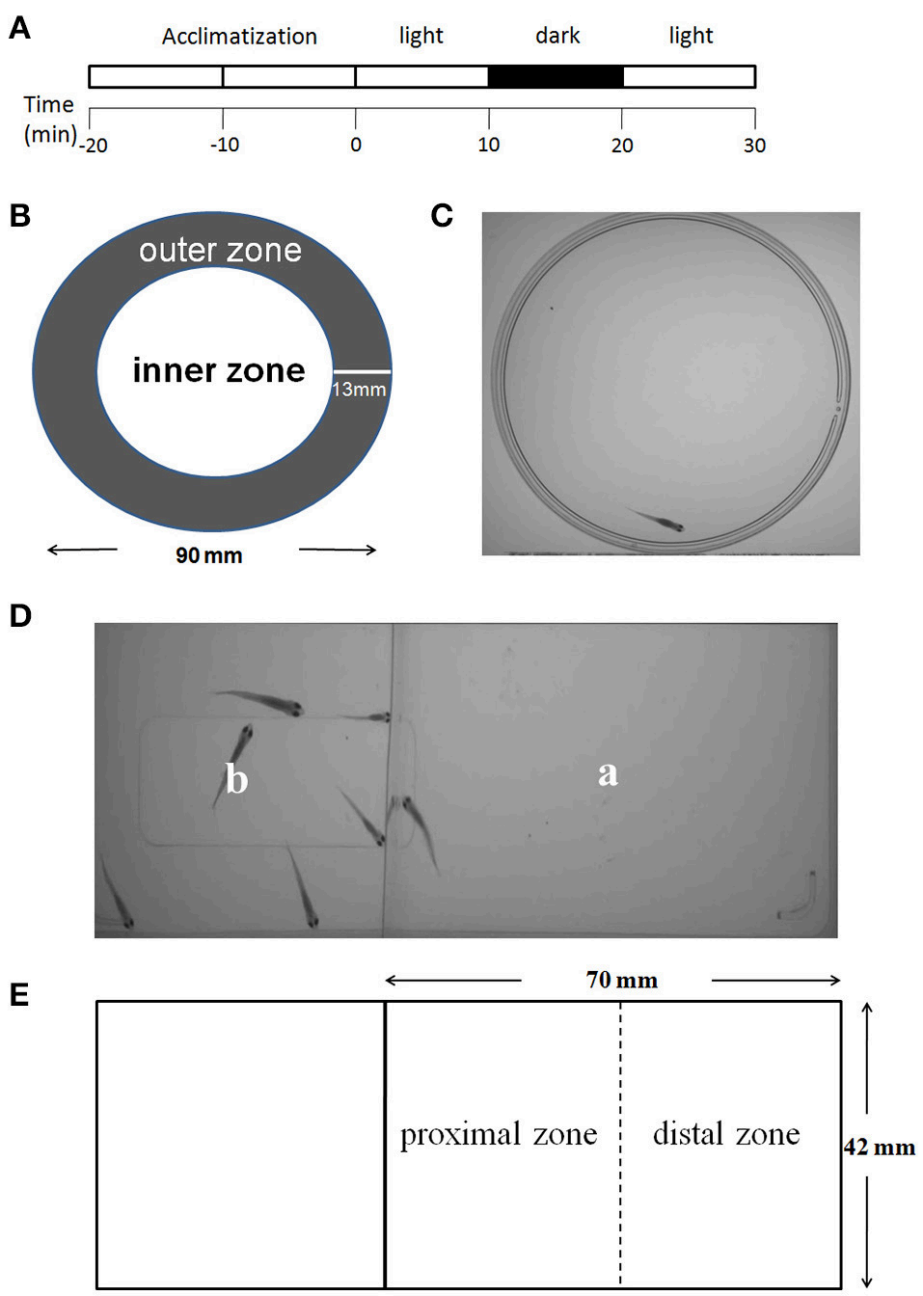
**Behavioral testing procedures**. The procedures for the behavioral tests of locomotor activity, thigmotaxis, and behavioral response to light change consisted of four steps: a 20 min acclimatization phase, the first light phase (minutes 0–10), a dark phase (minutes 10–20), and the second light phase (minutes 20–30) **(A)**. The tests of the above behaviors were performed in a dish (diameter of 90 mm). The inner and outer zones were delineated as shown above. The width of the outer zone was set at 13 mm to the border of the dish **(B,C)**. The social preference was tested in a rectangular chamber. The fish being tested was placed in area “a,” and six companion zebrafish were placed in area “b” **(D)**. “a” was the test area (70 × 42 mm) and was equally divided into one proximal zone and one distal zone **(E)**.

Behavioral tests were carried out with juvenile zebrafish at 1 month of age in dishes or rectangular chambers. All the experiments were performed 2 h after the beginning of the light cycle and 2 h before the beginning of the dark cycle. The experiments were arranged in a way that all groups were equally presented in dishes or rectangular chambers to avoid any inter-treatment variations due to differences in experiment timing during the day. The dish or rectangular chamber was then placed into a ZebraBox (ViewPoint Life Sciences) equipped with a recorder to record video of the juvenile zebrafish activities. The procedures for the behavioral tests are shown in Figure 2.

#### Locomotor activity

The quantification of zebrafish locomotor activity was achieved using the tracking mode of ZebraLab software with recorded videos. The videos of zebrafish were taken at 25 fps, and were pooled into 1 min time bins. Only the total distance traveled in the dish was obtained for analysis of the locomotor activity.

#### Thigmotaxis

A round center arena that occupied half of the area of the dish was defined in each dish (Figure 2B). Thigmotaxis was presented as the percentage (%) of the total distance moved (TDM) in the outer zone of the test apparatus as previously described by Schnörr et al. (2012). The percentage of TDM in the outer zone was obtained by multiplying this ratio by a factor of 100, as depicted in the formula below. This calculation was performed to correct for individual differences in locomotor activity, as recommended by Bouwknecht and Paylor (2008).

$$\begin{array}{c} \text{Thigmotaxis}\\ \left(\%\text{ TDM in outer zone}\right) \end{array}=\left[\frac{\text{TDM outer}}{\text{TDM outer}+\text{inner}}\right]\times 100$$

#### Social preference

A rectangular chamber was divided into “a” and “b” areas by a transparent barrier, so that animals in area “a” can see animals in area “b” (Figure 2D). The fish under test was placed in area “a,” and a six companion zebrafish were placed in area “b.” During analyses, area “a” was equally divided into one proximal zone and one distal zone (Figure 2E). Social preference was presented as the percentage (%) of the TDM or the percentage (%) of the total time spent (TTS) in the proximal zone of the test area. The percentage of TDM and TTS in the proximal zone was obtained by multiplying this ratio by a factor of 100 as depicted in the formula below. This calculation was performed to correct for individual differences in the locomotion activity.

$$\begin{array}{l} \begin{array}{c} \text{Social preference}\\ (\%\text{ TDM in }\\ \text{proximalzone}) \end{array}=\left[\frac{\text{TDM proximal}}{\text{TDM distal}+\text{proximal}}\right]\times 100\\ \begin{array}{c} \text{Social preference}\\ (\%\text{ TTS in }\\ \text{proximalzone}) \end{array}=\left[\frac{\text{TTS proximal}}{\text{TTS distal}+\text{proximal}}\right]\times 100 \end{array}$$

### Data presentation and statistics analysis

Data are presented as the mean ± SEM. Statistical analyses and graphs were performed using GraphPad Prism software (version 5.0).

One-way ANOVA followed by Dunnett's multiple-comparison *post-hoc* tests was employed to compare the VPA-treated groups with the controls to assess the effects of VPA on the locomotor activity, thigmotaxis, and social preference, and a probability level of 5% was used as the minimal criterion of significance.

Student's *t*-tests (two-tailed) were performed to analyze the behavioral changes in response to light change within each concentration group (light vs. dark). The minimal criterion of significance was set at 5%.

## Results

### Effects of chronic or acute exposure to VPA on social preference

**Figure 3:**
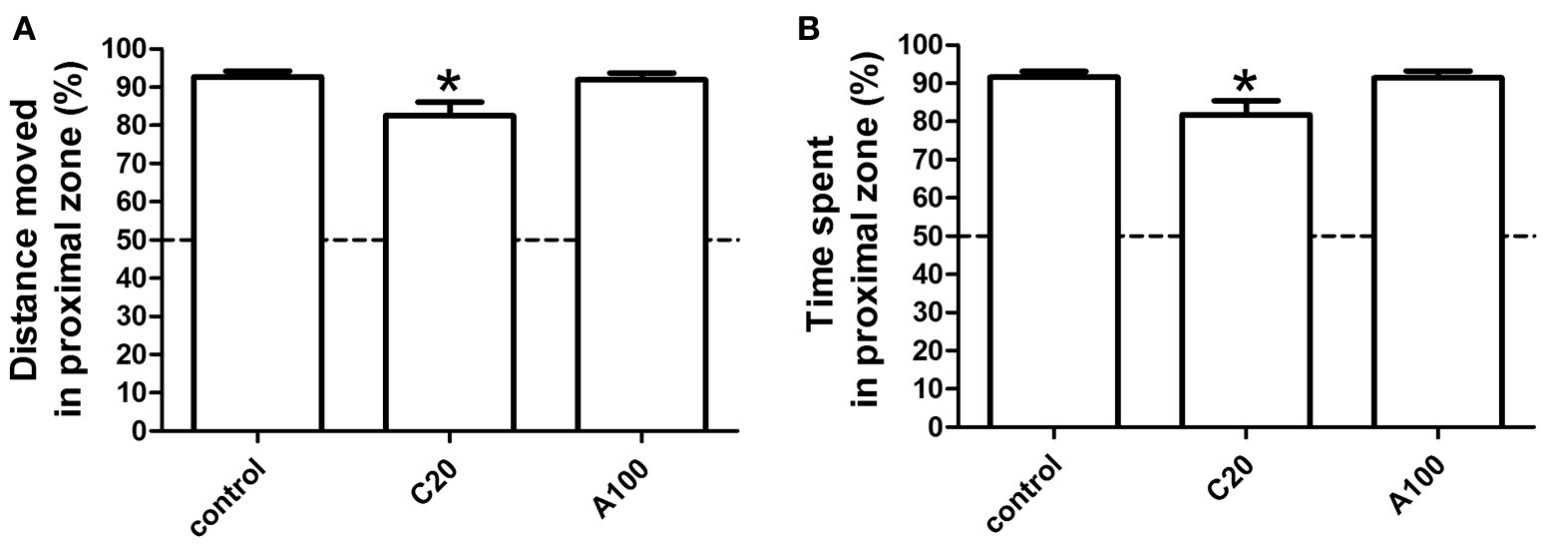
**Effects of chronic or acute exposure to VPA on social preference of zebrafish at 1 month of age**. The percentage of distance moved in the proximal zone during the first 10-min light phase was plotted in **(A)** and the percentage of time spent in the proximal zone during the first 10-min light phase (B) was plotted in **(B)**. The data are presented as the mean ± SEM, *n* = 32 animals per group. Statistical markers: ^*^p < 0.05; significantly different from the controls.

To test behaviors at 1 month of age, the concentration and the exposure window were set to maintain a fatality and deformity rate of < 50%. Two different but related parameters were defined to study social preference in zebrafish. One is the percentage (%) of the TDM in the proximal zone of the test area (%TMD in the proximal zone) and the other one is the percentage (%) of the TTS in the proximal zone of the test area (%TTS in the proximal zone). As shown in Figure 2E, the tested area “a” was divided into proximal and distal zones of equal size. Therefore, if no social preference was observed, %TMD in the proximal zone and %TST in the proximal zone would account for 50% of the activity in the whole area. It was obvious that in the non-treated groups, zebrafish demonstrated a clear social preference, as the %TMD in the proximal zone and %TST in the proximal zone were much more than 50% (Figure 3). Additionally, multiple comparisons in One-way ANOVA indicated that compared with the controls, chronic exposure to 20 μM VPA significantly decreased the %TMD in the proximal zone and %TTS in the proximal zone, while acute exposure to 100 μM did not significantly affect these parameters [*F*_(2, 116)_ = 5.332, Dunn's Method, *p* < 0.05; *F*_(2, 116)_ = 5.091, Dunn's Method, *p* < 0.05]. The results suggested that chronic exposure to 20 μM VPA significantly impaired social preference, while acute exposure to 100 μM VPA did not.

### Effects of chronic or acute exposure to VPA on the locomotor activity

**Figure 4:**
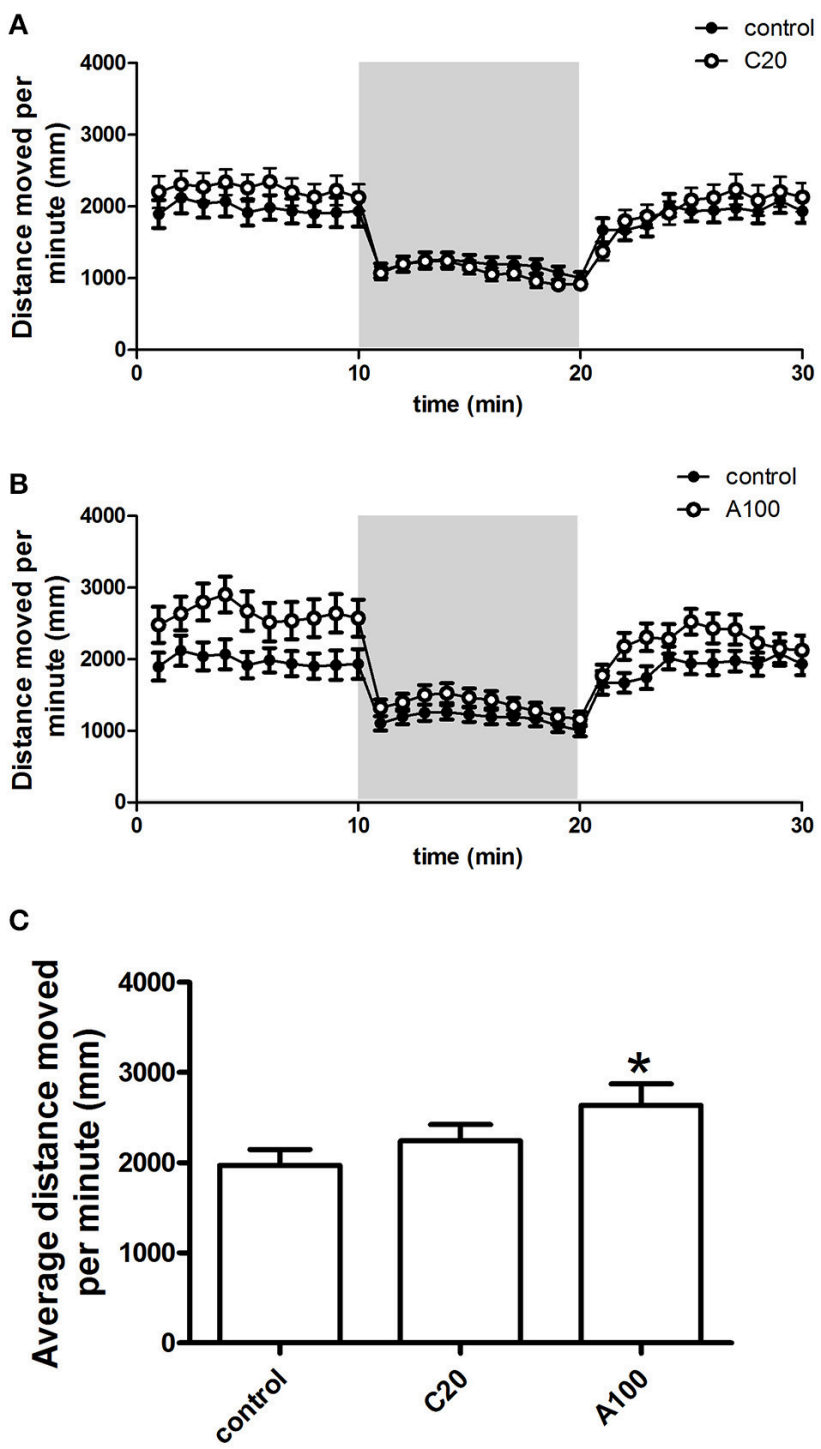
**Effects of chronic or acute exposure to VPA on the locomotor activity of zebrafish at 1 month of age**. Distance moved per minute in alternating 10-min blocks of light and dark. The control group (filled circles) was plotted with chronic exposure to 20 μM VPA (open circles) and acute exposure to 100 μM VPA (open circles) in **(A,B)**, respectively. The shaded part in each panel represents the dark phase, and the non-shaded part in each panel represents the light phase. Average distance moved during the first 10-min light phase was plotted in **(C)**. The data are presented as the mean ± SEM, *n* = 32 animals per group. Statistical markers: ^*^p < 0.05; significantly different from the controls.

The distance moved by zebrafish in each 1 min time bin during a 30 min period within the entire arena (petri dish, 90 mm in diameter) was plotted against the progression of the experiment, and the different VPA treatment groups were compared individually with the control group (Figures 4A,B). During the first light period, compared with the control group, increases in locomotor activity were observed in both VPA-treated groups, and the increases induced by acute VPA exposure were higher than those induced by chronic VPA exposure. During the subsequent dark period, the locomotor activity of both the controls and the VPA-treated groups remained at a relatively low level. During the second light period, the locomotor activity of the controls and the VPA-treated groups was similar to that in the corresponding groups during the first light period.

Detailed analysis was carried out to precisely evaluate the effects of the different VPA exposure procedures on the locomotor activity (Figure 4C). Average distance moved per minute by juvenile zebrafish during the first 10 min of the light condition was used to analyze the change in locomotor activity. As depicted above, multiple comparisons in One-way ANOVA indicated that compared with the controls, VPA exposure resulted in the increase of the locomotor activity, but only the acute exposure to 100 μM VPA produced a significant increase [*F*_(2, 105)_ = 2.759, Dunn's Method, *p* < 0.05].

### Effects of chronic or acute exposure to VPA on the thigmotaxis

**Figure 5:**
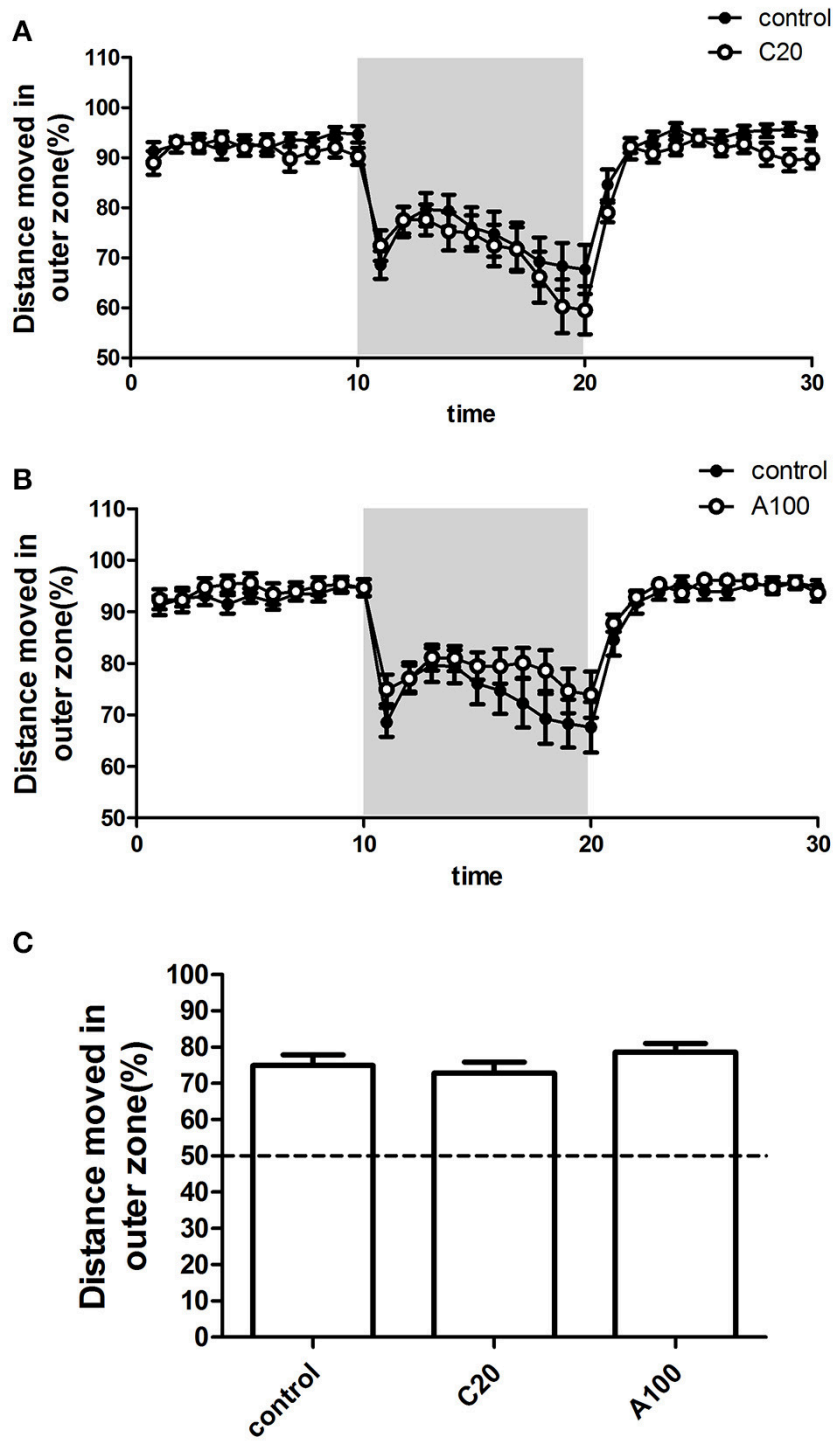
**Effects of chronic or acute exposure to VPA on thigmotaxis of zebrafish at 1 month of age**. Distance moved in outer zone (%) in alternating 10-min blocks of light and dark. The control group (filled circles) was plotted with chronic exposure to 20 μM VPA (open circles) and acute exposure to 100 μM VPA (open circles) in **(A,B)**, respectively. The shaded part in each panel represents the dark phase, and the non-shaded part in each panel represents the light phase. Average distance moved in the outer zone (%) during the 10-min dark phase was plotted in **(C)**. The data are presented as the mean ± SEM, *n* = 32 animals per group. Statistical markers: ^*^p < 0.05; significantly different from the controls.

The percentage (%) of the TDM in the outer zone of the test apparatus (%TMD in the outer zone) was measured as the level of thigmotaxis. %TMD in the outer zone in each 1 min time bin by zebrafish during the 30 min measurement period was plotted against the progression of the experiment, and the different VPA treatment groups were separately compared with the controls (Figures 5A,B). First, %TMD in the outer zone in all groups was >50%, suggesting that zebrafish in both control groups and VPA-treated groups exhibited thigmotaxis. During the first 10 min light phase, VPA-treated zebrafish demonstrated a level of thigmotaxis similar to that of the controls and all of them retained in a high level of thigmotaxis. During the beginning of the 10 min dark phase, VPA-treated zebrafish exhibited a level of thigmotaxis similar to that of controls, while during the latter part of the 10 min dark phase, compared with the controls, chronic exposure to 20 μM VPA resulted in a relatively low level of thigmotaxis, while acute exposure to 100 μM VPA resulted in a relatively high level of thigmotaxis. During the last 10 min light phase, VPA-treated zebrafish showed a high level of thigmotaxis similar to that of controls.

In addition, to analyze the effect of VPA on thigmotaxis, %TMD in the outer zone in the entire 10 min dark phase was calculated (Figure 5C). Multiple comparisons in One-way ANOVA indicated that compared with the controls, neither of the two VPA exposure procedures significantly affected the thigmotaxis [*F*_(2, 105)_ = 1.080, Dunn's Method, *p* = 0.3435].

### Effects of chronic or acute exposure to VPA on the zebrafish responses to sudden changes in light

The locomotor activity response of juvenile zebrafish to a sudden change in light was assessed as well. When the illumination suddenly switched from light to dark, the locomotor activity of controls and VPA-treated groups decreased immediately (Figures 4A,B). During the 10 min dark period, zebrafish continued to show low locomotor activity. When the illumination switched from dark to light, the locomotor activity of control groups and VPA treated groups increased immediately, and the activity level after the light change was similar to that observed during the first light period.

**Figure 6:**
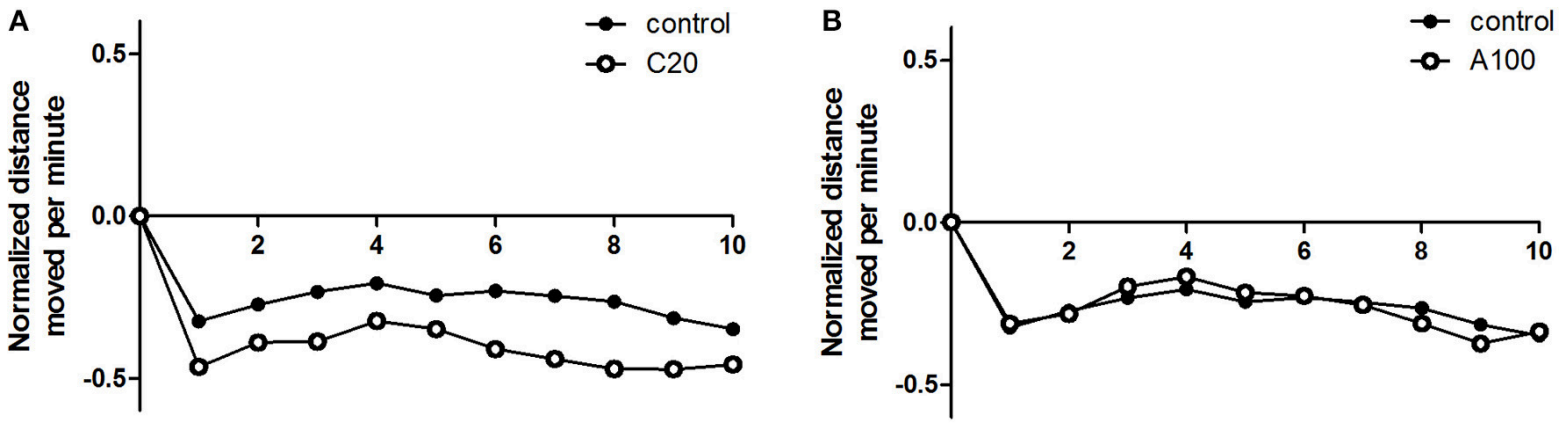
**Effects of chronic or acute exposure to VPA on behavioral responses to light change in zebrafish at 1 month of age**. Distance moved per minute in the 10-min dark phase was normalized against the average distance moved in the previous 10-min light phase. The control group (filled circles) was plotted with chronic exposure to 20 μM VPA (open circles) and acute exposure to 100 μM VPA (open circles) in **(A,B)**, respectively. The data are presented as the mean ± SEM, *n* = 32 animals per group.

As described above, sudden changes in lighting introduced changes in the locomotor activity in both the controls and the VPA-treated groups, and the response patterns were similar. Then, the locomotor activity during the 10 min dark period was analyzed to evaluate the effect of VPA exposure on zebrafish response to sudden changes in lighting. The locomotor activity during the dark period was normalized to the average locomotor activity during the first light period (Figure 6). Student's *t*-tests (two-tailed) indicated that compared with that in the controls, zebrafish with chronic exposure to 20 μM VPA showed a noticeable but not significant decrease in locomotor activity [*F*_(35, 35)_ = 1.728, *p* = 0.1103], and the relatively low locomotor activity was maintained during the entire 10 min dark phase, whereas zebrafish with acute exposure to 100 μM VPA showed locomotor activity similar to that of the controls.

## Discussion

In this study, two VPA treatment procedures, early chronic exposure to a low dose and early acute exposure to a high dose, each beginning at 24 hpf, were employed to analyze the neurobehavioral effects of VPA. Exposure to VPA before 24 hpf tended to result in much higher fatality and deformity rate than 50%, which was supported by the previous discovery that exposure to VPA beginning at gastrulation could affect common steps in the differentiation of multiple neuronal lineages (Jacob et al., 2014).

Zebrafish are a social species, but larvae do not exhibit the obvious shoaling and schooling observed in adults. Social preference is a common behavior in which animals prefer to be near their conspecifics and it is prevalent among social animals. Dreosti et al. found that zebrafish did not exhibit overt social preference until 3 weeks of age (Dreosti et al., 2015), while our work suggested that social preference was not observable until 4 weeks of age (data not shown). The differences between these results could be attributed to different experimental conditions, such as different experimental chambers. Thus, in this work, behavioral tests were conducted at 1 month of age.

VPA is a widely used medicine for treating or preventing epilepsy, migraine, and bipolar disorder. However, VPA administration during pregnancy may result in fetal valproate syndrome which has features similar to autism, such as impaired social behavior (Ardinger et al., 1988; Koch et al., 1996; Moore et al., 2000; Mawer et al., 2002). The behaviors of rats exposed to VPA *in utero* have been examined by researchers (Rodier et al., 1996; Schneider and Przewlocki, 2005; Markram et al., 2008; Schneider et al., 2008). The effects of VPA on animals' behavior are closely related to the exposure window and the concentration. Kim et al. have compared the effects of different time windows of prenatal VPA exposure on social behavior in Sprague–Dawley rats and found that compared with exposure at E7, E9.5, or E15, VPA exposure at E12 produced significantly reduced sociability and social preference (Kim et al., 2011). A similar phenomenon was observed by Kataoka et al. (2013). Bailey compared the effects of different concentrations of VPA on zebrafish behaviors and found that compared with exposure to 0.5, 15, 30, and 50 μM from 4 to 5 dpf, exposure to 5 μM VPA from 4 to 5 dpf impaired social behavior in adult zebrafish (Bailey et al., 2015). In this study, we analyzed the effects of VPA on zebrafish behaviors using two different exposure procedures and found that early chronic exposure to VPA at low concentration significantly reduced social preference in juvenile zebrafish, whereas acute exposure to VPA at a high concentration did not significantly affect social preference. Our results suggested that the effect of VPA on social behavior was closely relative to the exposure procedure, and further work needs to be done to clarify whether there are sensitive exposure windows and exposure concentrations for modifying social behaviors in zebrafish and to identify those time windows and concentrations if they exist.

Previous studies demonstrated that developmental exposure to VPA at 15 μM from 4 to 5 dpf significantly increased the locomotor activity at 6 dpf (Bailey et al., 2015), our results indicated that this stimulatory effect of VPA treatment on zebrafish locomotor activity may not be sustained, given that chronic exposure to 20 μM VPA from 24 to 6 dpf (7 h per day) did not significantly affect the locomotor activity at 30 dpf. Acute larval exposure to 100 μM VPA from 24 to 31 hpf significantly increased the locomotor activity, while exposure to VPA at 48 μM during the first 48 hpf did not significantly modify the locomotor activity at 30 dpf (Zimmermann et al., 2015), implying that the effects of VPA on the locomotor activity depended on both exposure concentration and exposure window. The results from both the current work and previous work suggested that 24–32 hpf may be a sensitive window for modification of the locomotor activity of juvenile zebrafish and that exposure concentration also plays a vital role as well.

In summary, our results showed that chronic exposure to low concentration of VPA impaired social preference without changing the locomotor activity, anxiety, and light-response behaviors, implying that the perceived social behavior change is not due to changes in other simple behaviors, and that this would be a suitable model for analyzing the mechanism underlying the effect of VPA on social behavior.

## Conclusion

The present study aimed to evaluate the effects of VPA on simple and complex behaviors of juvenile zebrafish with two different exposure procedures. The behavioral analysis showed that chronic exposure to VPA at a low concentration impaired social preference while exerting no effect on other simple behaviors, such as the locomotor activity, anxiety, and behavioral responses to light change. However, among the tested behaviors, acute exposure to VPA at high concentration only significantly increased the locomotor activity. In conclusion, the neurobehavioral effects of VPA depended on both exposure concentration and exposure window.

## Author contributions

All listed authors have made significant contributions to this work, and submission of this manuscript for publication has been approved by all of the authors.

### Conflict of interest statement

The authors declare that the research was conducted in the absence of any commercial or financial relationships that could be construed as a potential conflict of interest.
